# Race and “omic” data in glioma: A systematic review of contemporary research to explore the digital divide

**DOI:** 10.1093/nop/npaf016

**Published:** 2025-01-31

**Authors:** Olaoluwa Ezekiel Dada, Zvipo Chisango, Kwadwo Antwi Boasiako Nkansah-Poku, Mareshah N Sowah, Amanda Cyntia Lima Fonseca Rodrigues, Olivia Duru, Matthew Myers, Sophie T Williams, Shungu Ushewokunze, Spencer J Collis, Nathan A Shlobin, Sylvester Omoruyi, Ola Rominiyi

**Affiliations:** College of Medicine, University College Hospital Ibadan, Ibadan, Oyo State, Nigeria; Emory University School of Medicine, Atlanta, Georgia, USA; College of Health Sciences, University of Ghana Medical School, College of Health Sciences, Accra, Ghana; University of South Carolina School of Medicine Greenville, Greenville, South Carolina, USA; Department of Neurology, University of North Carolina at Chapel Hill, Chapel Hill, North Carolina, USA; The Ohio State University College of Medicine, Columbus, Ohio, USA; Department of Neurosurgery, Sheffield Teaching Hospitals NHS Foundation Trust, Sheffield, UK; Weston Park Cancer Centre, Sheffield Teaching Hospitals NHS Foundation Trust, Sheffield, UK; Division of Clinical Medicine, University of Sheffield, Sheffield, UK; Department of Neurosurgery, Sheffield Teaching Hospitals NHS Foundation Trust, Sheffield, UK; Division of Clinical Medicine, University of Sheffield, Sheffield, UK; Department of Neurosurgery, Neurological Institute of New York, Columbia University Irving Medical Center, New York, New York, USA; School of Anatomical Sciences, Faculty of Health Sciences, University of the Witwatersrand, Johannesburg, South Africa; Division of Neuroscience, University of Sheffield, Sheffield, UK; Division of Clinical Medicine, University of Sheffield, Sheffield, UK; Department of Neurosurgery, Sheffield Teaching Hospitals NHS Foundation Trust, Sheffield, UK

**Keywords:** diversity and inclusion (EDI), equality, ethnicity, glioma, omics, race

## Abstract

**Background:**

The expanding repertoire of studies generating genome-scale omic datasets from glioma samples provides a generational opportunity to uncover mechanisms driving aggressive biology and develop new treatments. However, ensuring such studies reflect the breadth of racial groups and ethnicities affected by gliomas is critical to support equity in future therapeutic advances. We therefore report a contemporary snapshot of the representation of race and ethnicity in omic glioma studies.

**Methods:**

We searched PubMed, Embase, Web of Science, and Scopus and systematically reviewed articles published between January and November 2023 reporting de novo genome-scale sequencing data generated using samples from patients diagnosed with glioma (according to World Health Organization 2021 criteria) to characterize the reporting and composition of race and ethnicity data.

**Results:**

Thirty-five studies involving 5601 patients were analyzed. Race or ethnicity data was reported in only 3 studies (8.6%), of which none provided omic data in a format that could be stratified by race or ethnicity. Reporting varied by continent with all 3 studies including race or ethnicity data based in North America. Where racial data was available, we found that samples used for genome-scale characterization came from patients reported as being White in 91.1% cases (41 patients), with 6.7% (3 patients) reported as Black and 2.2% (1 patient) as Hispanic.

**Conclusions:**

These studies underscore an urgent need for improved reporting and representation to enhance our understanding of glioma biology across different populations and guide targeted initiatives from policymakers and funders to support equitable improvements in healthcare.

## Graphical Abstract

**Graphical Abstract:**
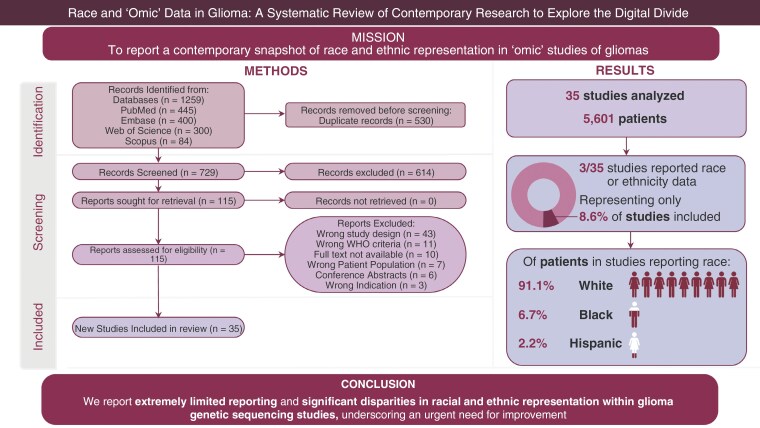

**Key Points**

- Recent glioma studies generating omic data often exclude race/ethnicity information.
- Where available, Black, Hispanic, and sub-Saharan African representation is lacking.
- Routine reporting of race and ethnicity is key to equitable healthcare advancement.

**Importance of the Study**

Like many other tumors, the genetic variations present in gliomas may differ among racial and ethnic groups. These variations could impact the risk, progression, and treatment of gliomas. As such, this study highlights the disparities in representation within genomic studies on gliomas, which are critical in developing more effective and personalized treatments for glioma patients. This study highlights profound under reporting and reiterates the importance of capturing racial and ethnic data in databases to improve data reliability, generalizability, inclusivity, and social justice. Importantly, identifying potential variations in the genetic signatures of gliomas between races and ethnic groups will help optimize context-specific therapeutic strategies and improve precision medicine for all.

## Background

Tumors of the central nervous system (CNS) are an important contributor to morbidity and mortality with an estimated 330 000 tumors diagnosed each year globally.^1^ Of these, gliomas are the most common primary brain tumors of the CNS, with data from the United States reporting that 26.3% of all CNS tumors are gliomas.^2^ In adults, these tumors typically range from lower-grade gliomas (World Health Organization [WHO] grade 2) to more aggressive high-grade gliomas (WHO grade 3–4) and are increasingly described, and often even defined, on the basis of detailed molecular characteristics.^3^ Specific genetic and epigenetic features in gliomas can influence prognosis as well as predict the potential benefits of particular treatments. For example, the clinical benefit of treatment using the alkylating chemotherapeutic temozolomide in glioblastoma is almost exclusively associated with hypermethylation of the O^6^-methylguanine DNA methyltransferase (MGMT) gene promoter.^4,5^ While more recently, drugs targeting neomorphic mutation of the isocitrate dehydrogenase (IDH) enzymes IDH1 and IDH2 have been demonstrated to deliver target-specific biological activity and improve progression-free survival (PFS) in lower-grade gliomas.^6,7^ It has also been shown that there are differences in the incidence of genetic variants by racial group^8^ as well as differences in the survival of patients diagnosed with glioblastoma among different ethnicities. For example, it has been found that incidence and mortality for glioma are higher in non-Hispanic Whites than in Hispanic Whites.^9^ It has also been found that increased European ancestry in some Black American and Hispanic populations is associated with increased risk of glioma.^10^

The concepts of race and ethnicity are interrelated but have distinct meanings. Race is based on the physical observations that Europeans had of different populations. Therefore, race is primarily based on physical characteristics such as skin, hair, and eye color. Ethnicity, on the other hand, is more centered around cultural features including language, ancestry, religion, and for some groups, shared nationality.^11^ According to the United States Census Bureau, 58.4% of the US population identified as non-Hispanic White, 19.5% as Hispanic, 13.7% as Black, and 6.4% as Asian.^12^ The racial makeup of Europe varies between nations. In the United Kingdom estimates from 2021 suggest 81.7% of the population in England and Wales were White, 9.3% were Asian, 4.0% were Black, 2.9% were Mixed, and 2.1% identified as “Other.”^13^ Reliable statistics detailing the overall racial composition of Africa are also difficult to obtain. In South Africa, one of the most racially diverse African nations, 81.4% of the population identify as Black African, 8.2% as Colored (a term used in southern Africa to describe people with mixed heritage/ancestries), 7.3% as White, 2.7% as Indian/Asian, and 0.4% identified as Other.^14^

Despite an understanding that knowledge of the specific mutations in glioma can advance care, previous genome-scale sequencing projects have overrepresented patients from some racial and ethnic backgrounds and underrepresented others. For example, the landmark 2008 TCGA (The Cancer Genome Analysis) study that provided detailed genome-scale characterization of tumors from 206 glioblastoma patients included 81 White (39.3%) patients and only 2 Black (1.0%), 1 Asian (1.0%), and 1 American Indian (1.0%) patients, with no race associated race data available for the majority of patients.^15^ Other examples include the Ivy Glioblastoma Atlas Project (GAP), which examined the clinical and molecular features of samples from 41 patients in the context of histologically defined anatomical tumor regions but did not report the racial breakdown of patients included.^16^ Similarly, The Glioma Longitudinal AnalySiS (GLASS) Consortium, a major longitudinal undertaking looking at the evolution of glioma molecular profiles from initial occurrence to recurrence, also did not report data on the ethnicity and race of patients providing samples for sequencing studies.^17,18^ A more recent assessment of 4 genome banks from TCGA, the Therapeutically Applicable Research to Generate Effective Treatments (TARGET), cancer-related genome-wide association study (GWAS), and the OncoArray Consortium demonstrated potentially unrepresentative sampling with 91.1% of total samples coming from White patients.^19^ In summary, several large studies substantially underrepresented some racial and ethnic groups, while many other large studies have not reported ethnic or racial data for their participants. This makes it difficult to assess possible differences in the molecular characteristics of gliomas, and any potential therapeutic or prognostic impact, between distinct racial or ethnic groups. Initiatives through consortia led from outside of North America and Europe, such as the Chinese Glioma Genome Atlas (CGGA), have made some progress towards redressing the underrepresentation of specific ethnicities in glioma sequencing efforts.^20^ However, it is possible that many racial and ethnic groups remain substantially underrepresented, and a paucity of genome-scale data characterizing gliomas within sub-Saharan African populations persists in the literature.

As omic sequencing technologies continue to evolve, decrease in cost (in some cases), and become more widely used, the rapidly expanding repertoire of genome-scale datasets available to researchers provides a generational opportunity to deliver step-changes in our understanding of glioma biology and new, more effective therapeutic approaches. However, since profound intra-tumoral and inter-patient heterogeneity are well-established characteristics of gliomas^21,22^ there is a concerning potential for a lack of representation of specific patient groups within such omic datasets to portend uncertainty around the efficacy of new treatments in these same groups and further entrench existing inequalities in healthcare outcomes. Specifically, considering previously reported differences in molecular features and survival rates for multiple types of cancer (including non-CNS cancers) among racial and ethnic groups,^9,10^ there is an important and urgent need to establish whether current reporting of race and ethnicity data in contemporary studies generating new genome-scale sequencing data for gliomas is adequate. It is also important to determine whether the distribution of research samples used closely reflects the ethnic and racial composition of wider populations at a national and international level. The aim of this systematic review is to therefore provide a snapshot describing the current state of racial and ethnic representation in recently published studies which have generated de novo genome-scale sequencing data from glioma samples.

## Method

### Study Design

**Figure 1.**
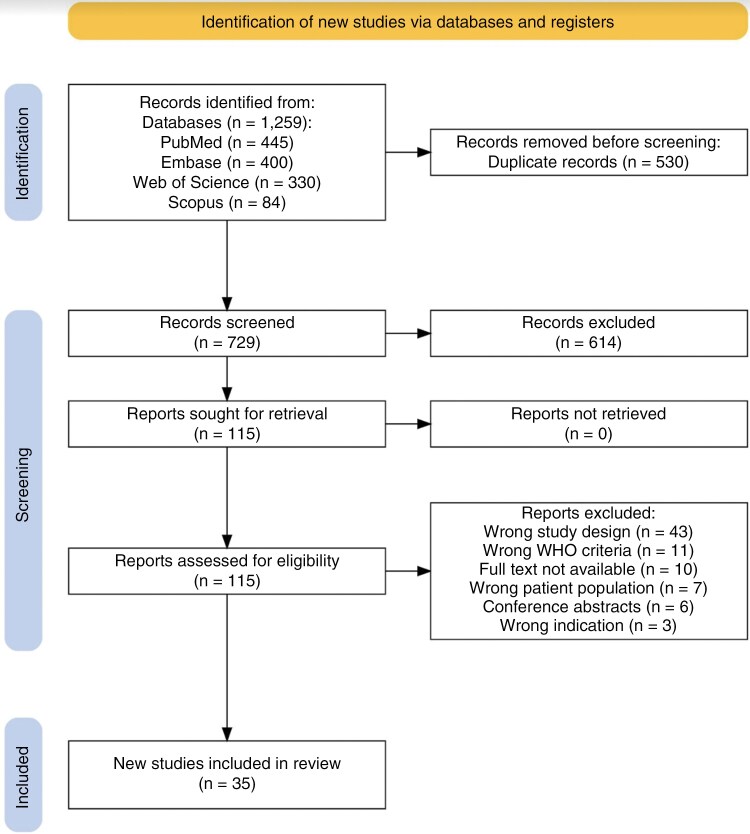
Identification and screening of glioma studies including de novo genome-scale sequencing data.

This systematic review was performed and reported in line with the Preferred Reporting Items for Systematic Review and Meta-Analysis (PRISMA) guidelines^23^ (Figure 1).

### Search Strategy

Although the most recent WHO classification of CNS tumors was published in 2021, we anticipated that it would take at least 1–2 years or more for relevant omic studies using the most recent classification to begin to be published. Therefore, PubMed, Embase, Web of Science, and Scopus were systematically searched for studies published between January 1, 2023 and November 11, 2023 (the date of our initial searches), to provide a contemporary snapshot of publications using the WHO 2021 classification. A comprehensive search strategy was developed to identify articles with new genome-scale sequencing data for gliomas using the Medical Subject Heading terms and Boolean operators described in the Supplementary Table. The search strategy was modified and adapted to other databases identified above.

### Selection Criteria

New studies with the generation of de novo genome-scale/omic data (ie, excluding studies which only re-analyze previously published datasets) for patient-derived specimens including from tumor tissue, patient-derived cell lines, or liquid biopsy for adult patients diagnosed with a diffuse glioma were included. Datasets considered to be genome-scale/omic included whole-genome sequencing (WGS), whole-exome sequencing (WES), RNA sequencing (RNAseq), methylation sequencing techniques (including ATACseq), in addition to targeted gene panels or proteomics approaches assessing more than 20 genes/proteins (Table 1). However, studies reporting both adult and pediatric data were only included if it was possible to stratify the data, to enable an isolated analysis of the adult cases only. We excluded studies making use of data from previously published, publicly available databases as a primary source for their data. Studies that include both new omic data and data from public databases without stratifying the data were excluded in order to avoid over/duplicate reporting of findings and avoid including ineligible patients.

**Table 1.** Definition of Key Terms

Terms	Definition
Race	*Any one of the groups that humans are often divided into based on physical traits regarded as common among people of shared ancestry* (Merriam-Webster).
Ethnicity	*Of, or relating to, large groups of people classed according to common racial, national, tribal, religious, linguistic, or cultural origin or background* (Merriam-Webster).
Omic data	Data obtained from high throughput technology to measure biological molecules such as the genome or the transcriptome.
Public database	A set of data available for public use.

Due to the nature of this study, randomized clinical trials (RCTs), basic science studies, original research, case-controlled and cohort observational studies, and pre-prints were eligible for inclusion. Book chapters, scoping and systematic reviews, conference papers, all studies utilizing pre-2021 WHO classifications, pediatric studies, and studies written in any language other than English were excluded.

### Data Management

Data records were downloaded from the above databases and managed through COVIDENCE systematic review software (*Veritas Health Innovation*) to filter duplicates and screen titles, abstracts, and full text. This was done in line with our outlined eligibility criteria. Full-text screened articles were exported to Microsoft Excel (Microsoft). A proforma was made on Microsoft Excel for full-text data extraction and collation of final datasets.

### Study Selection

The title and abstract of each study were screened by 2 independent authors using the predefined eligibility criteria. Potentially eligible studies were then subjected to full-text screening by 2 independent reviewers using the eligibility criteria, including screening of supplementary materials where required. At both stages of screening, conflicts were discussed between the 2 reviewers and, where required, the senior author to help establish a consensus.

### Data Extraction

Full-text screened articles were exported into a data extraction proforma on Microsoft Excel (Microsoft). Data was extracted on: (a) study design, (b) study demographics, (c) country of origin, (d) tumor histopathology, (e) tumor grade, (f) race, (g) ethnicity, (h) astrocytoma (IDHmut), (i) oligodendroglioma, and (j) glioblastoma.

### Risk of Bias and Quality Assessment

**Figure 2.**
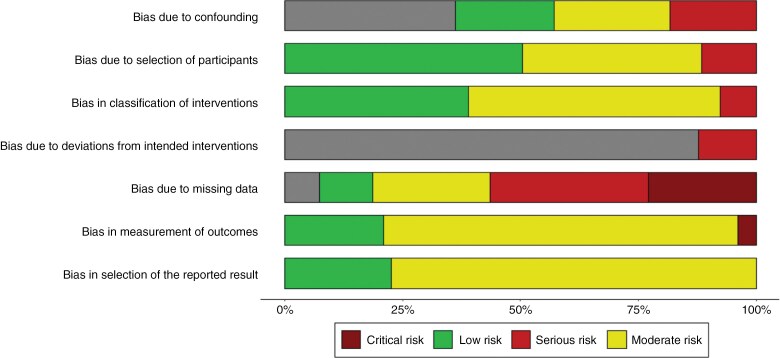
Risk of bias assessment summary.

The Cochrane Risk Of Bias In Non-randomized Studies-of Intervention (ROBINS-I) tool was used to assess the risk of bias in the included studies. Bias due to confounding, in selection of participants, in classification of interventions, due to deviation from intervention, due to missing data, in measurement of outcomes, and in selection of reported outcomes were specific domains assessed in each study using the ROBIN-I tool. The overall domain-level judgment within the ROBINS-I tool was rated 0 = “No” information; 1 = “No”; 2 = “Probably no”; 3 = “Probably yes”; and 4 = “Yes.” Afterwards, an overall bias for each study was defined as 0 = “No information”; 1 = “Low risk of bias”; 2 = “Moderate risk of bias”; 3 = “Serious risk of bias”; and 4 = “Critical risk of bias” (see Figure 2).

The quality of each study was assessed through the Grading of Recommendations Assessment, Development, and Evaluation (GRADE) approach.^24^ Grading of recommendations assessment, development, and evaluation criteria are used to evaluate the certainty of evidence and strength of recommendations. The certainty of evidence was reported as high, moderate, or low using 5 criteria for downgrading (risk of bias, inconsistency, imprecision, publication bias, and indirectness) and 3 criteria for upgrading (a large effect, dose-response, and residual confounding opposing the observed effect). Certainty, as determined using GRADE criteria, indicates the level of confidence we can have in the conclusions of the study and the likelihood that more research on the topic would change the conclusions. Grading of recommendations assessment, development, and evaluation assessments are reported in a summary table of each study included in our analysis (Table 2).

**Table 2.** Details of 35 Studies Eligible for Review of Race and Ethnicity Data Reporting

Journal	Study title	Country of study	Continent of study	Number of glioma patients	Type of technique used	Study period	Grade	Overall bias	Report race/ethnicity
Iwahashi et al.^25^ (*Journal of Neurosurgery*)	2-Hydroxyglutarate magnetic resonance spectroscopy in adult brainstem glioma	Japan	Asia	10	Methylation profiling, gene panel	2005–2022	Low	Moderate	No
Won Choi et al.^26^ (*Cancer Medicine*)	A multicenter, phase II trial of GC1118, a novel anti-EGFR antibody, for recurrent glioblastoma patients with *EGFR* amplification	Republic of Korea	Asia	21	Whole-exome sequencing, RNA sequencing	2018–2020	Moderate	Critical	No
Muench et. al.^27^ (*The American Journal of Surgical Pathology*)	A novel type of IDH wild-type glioma characterized by gliomatosis cerebri-like growth pattern, TERT promoter mutation, and distinct epigenetic profile	Germany	Europe	16	Whole-exome sequencing, genome-wide methylation profiling	NA	Low	Serious	No
Makino et al.^28^ (*Neuro-Oncology Advances*)	Alterations in EGFR and PDGFRA are associated with the localization of contrast-enhancing lesions in glioblastoma	Japan	Asia	124	Gene panel, methylation profiling	NA	High	Moderate	No
Zappe et. al.^29^ (*Cells*)	Association between *MGMT* enhancer methylation and *MGMT* promoter methylation, MGMT protein expression, and overall survival in glioblastoma	Austria	Europe	40	Methylation profiling	2001–2020	Moderate	Moderate	No
Galbraith et al.^30^ (*Neuro-Oncology Advances*)	Clinical utility of whole-genome DNA methylation profiling as a primary molecular diagnostic assay for central nervous system tumors—a prospective study and guidelines for clinical testing	USA	North America	606	Methylation profiling, DNA and RNA sequencing	2014–2022	High	Serious	No
Kahng et al.^31^ (*Journal of the European Society for Therapeutic Radiology and Oncology*)	Clinicogenetic characteristics and the effect of radiation on the neural stem cell niche in subventricular zone-contacting glioblastoma	Republic of Korea	Asia	125	Gene panel	NA	Low	Moderate	No
Carlos-Escalante et al.^32^ (*Journal of Neuro-Oncology*)	Deep DNA sequencing of MGMT, TP53, and AGT in Mexican astrocytoma patients identifies an excess of genetic variants in women and a predictive biomarker	Mexico	North America	48	Gene panel, methylation profiling	2013–2022	Low	Serious	Yes
Ofek et al.^33^ (*International Journal of Cancer*)	Deoxyhypusine hydroxylase: a novel therapeutic target differentially expressed in short-term versus long-term survivors of glioblastoma	Israel	Asia	87	RNA sequencing, proteomics	NA	Moderate	Moderate	No
Zhang et al.^34^ (*Nature Portfolio Journals, Precision Oncology*)	Distinct aneuploid evolution of astrocytoma and glioblastoma during recurrence	China	Asia	65	Whole-exome sequencing	2000–2018	Moderate	Low	No
Jackson et al.^35^ (*BioRxiv*)	Distinct myeloid-derived suppressor cell populations promote tumor aggression in glioblastoma	USA	North America	33	RNA sequencing	NA	Moderate	Moderate	No
Higa et al.^36^ (*Cancer Medicine*)	Distribution and favorable prognostic implication of genomic EGFR alterations in IDH-wild-type glioblastoma	Japan	Asia	138	Gene panel, methylation profiling	2014–2021	High	Serious	No
Mut et al.^37^ (*Cancer*)	Extracellular-vesicle-based Cancer panels diagnose glioblastomas with high sensitivity and specificity	Turkey	Eurasia*	116	RNA sequencing	NA	High	Critical	No
Higa et al.^38^ (*Neuro-Oncology Advances*)	Favorable prognostic impact of phosphatase and tensin homolog alterations in wild-type isocitrate dehydrogenase and telomerase reverse transcriptase promoter glioblastoma	Japan	Asia	208	Gene panel	2014–2022	High	Serious	No
Lazzarini et al.^39^ (*Journal of Neuro-Oncology*)	Genome-wide profiling of patient-derived glioblastoma stem-like cells reveals recurrent genetic and transcriptomic signatures associated with brain tumors	Italy	Asia	94	Whole-exome sequencing	NA	High	Moderate	No
Brawanski et al.^40^ (*International Journal of Molecular Sciences*)	Influence of MMR, MGMT promotor methylation, and protein expression on overall and progression-free survival in primary glioblastoma patients treated with temozolomide	Austria	Europe	42	Methylation profiling	2015–2018	Moderate	Serious	No
Orzan et al.^41^ (*Clinical Cancer Research*)	Liquid biopsy of cerebrospinal fluid enables selective profiling of glioma molecular subtypes at first clinical presentation	Italy	Europe	84	Gene panel, methylation profiling, fluorescence in situ hybridization	NA	Moderate	Serious	No
Otsuji et al.^42^ (*Neuro-Oncology Advances*)	Liquid biopsy with multiplex ligation-dependent probe amplification targeting cell-free tumor DNA in cerebrospinal fluid from patients with adult diffuse glioma	Japan	Asia	25	Gene panel	2019–2022	Moderate	Moderate	No
DalBo et al.^43^ (*Journal of Translational Medicine*)	Machine learning to improve interpretability of clinical, radiological, and panel-based genomic data of glioma grade 4 patients undergoing surgical resection	Italy	Europe	102	Gene panel	2014–2019	Low	Moderate	No
Benusiglio et al.^44^ (*Cancer Genetics*)	Mismatch repair deficiency and Lynch syndrome among adult patients with glioma	France	Europe	1225	Gene panel	2017–2022	Moderate	Serious	No
Liu et al.^45^ (*iScience*)	Molecular and clonal evolution in vivo reveal a common pathway of distant relapse gliomas	China	Asia	52	Whole-exome sequencing	NA	Low	Moderate	No
Romanidou et al.^46^ (*Oncology Letters*)	Molecular profile and clinical features of patients with gliomas using a broad targeted next-generation-sequencing panel	Greece	Europe	32	Gene panel	NA	Low	Serious	No
Svensson et al.^47^ (*Neuro-Oncology Advances*)	MR elastography identifies regions of extracellular matrix reorganization associated with shorter survival in glioblastoma patients	Norway	Europe	13	RNA sequencing	NA	Moderate	Moderate	No
Carenza et al.^48^ (*Frontiers in Immunology*)	Perioperative corticosteroid treatment impairs tumor-infiltrating dendritic cells in patients with newly diagnosed adult-type diffuse gliomas	Italy	Europe	27	RNA sequencing	NA	Low	Moderate	No
Kessler et al.^49^ (*Clinical Cancer Research*)	Prognostic markers of DNA methylation and next-generation-sequencing in progressive glioblastoma from the EORTC-26101 trial	Germany	Europe	380	Gene panel, methylation profiling	2011–2015	High	Critical	No
Jiménez et al.^50^ (*Neuropathology*)	Quantitative analysis of MGMT promoter methylation status changes by pyrosequencing in recurrent glioblastoma	Spain	Europe	24	Methylation profiling	2009–2021	Low	Critical	No
Ghosh et al.^51^ (*Science Translational Medicine*)	Radiation-induced circulating myeloid-derived suppressor cells induce systemic lymphopenia after chemoradiotherapy in patients with glioblastoma	USA	North America	20	RNA sequencing	NA	Moderate	Serious	Yes
Kumar et al.^52^ (*Journal of Molecular Neuroscience*)	RNA sequencing of intraoperative peritumoral tissues reveals potential pathways involved in glioma-related seizures	India	Asia	12	RNA sequencing	NA	Low	Serious	No
Kapteijn et al.^53^ (*Thrombosis Research*)	RNA sequencing to discover genes and signaling pathways associated with venous thromboembolism in glioblastoma patients: a case-control study	Netherlands	Europe	23	RNA sequencing	2017–2020	Low	Critical	No
Braun et al.^54^ (*Cells*)	Scaffold-based (Matrigel) 3D culture technique of glioblastoma recovers a patient-like immunosuppressive phenotype	Germany	Europe	58	RNA sequencing, methylation profiling	NA	Moderate	Moderate	No
Buyuktepe et al.^55^ (*Journal of Neuro-Oncology*)	Significance of O^6^-methyl guanine methyltransferase promoter methylation in high-grade glioma patients: optimal cutoff point, CpG locus, and genetic assay	Turkey	Eurasia*	95	Methylation sequencing	2008–2019	Low	Serious	No
Kapteijn et al.^56^ (*Thrombosis Research*)	Targeted DNA sequencing to identify genetic aberrations in glioblastoma that underlie venous thromboembolism; a cohort study	Netherlands	Europe	328	Gene panel	2017–2020	High	Critical	No
Villani et al.^57^ (*Journal of Translational Medicine*)	The glioma-IRE project—molecular profiling in patients with glioma: steps toward an individualized diagnostic and therapeutic approach	Italy	Europe	99	Gene panel	2019–2022	Moderate	Critical	No
Qi et al.^58^ (*Nature Portfolio Journal*)	Transcriptomic analyses of patient peripheral blood with hemoglobin depletion reveal glioblastoma biomarkers	USA	North America	15	RNA sequence	NA	High	Serious	Yes
McCord et al.^59^ (*Acta Neuropathologica Communications*)	Variant allelic frequencies of driver mutations can identify gliomas with potentially false-negative MGMT promoter methylation results	USA	North America	658	Methylation profiling	2006–2022	Moderate	Moderate	No

^*^Classified as Asia for subsequent continent groupings within this study.

### Data Analysis

Descriptive statistics were used to summarize the demographic and clinical characteristics. Frequencies and percentages were used for categorical variables, such as ethnicity and race reporting. All analyses were conducted using Python with pandas, NumPy, SciPy, and matplotlib libraries. The risk of bias assessment stacked bar chart was generated using R software (R version 4.4.1).

## Results

### Study Demographics

Thirty-five studies were included in our analysis with a total of 5601 patients collectively. The mean age was 56.7 (±6.2 SD) years. With respect to sex, 44.0% (2457 patients) were male, while 30.1% (686 patients) were female, and 26.0% (1458 patients) did not have their gender specified.

### Tumor Distribution by Grade

A total of 33 (94.3%) studies analyzed reported grades of studied glial tumors. In those studies, 0.3% were grade 1 tumors, 3.9% were grade 2 tumors, 3.1% were grade 3 tumors, and 92.6% were grade 4 tumors (Table 3).

**Table 3.** Glial Tumors With Reported Grade

Variable	Level	*N* (%)
Tumor type by grade	Grade 1 tumors	10 (0.3)
	Grade 2 tumors	155 (3.9)
	Grade 3 tumors	121 (3.1)
	Grade 4 tumors	3599 (92.6)
Type of glioma	Astrocytoma	516 (11.9)
	Oligodendroglioma	249 (5.8)
	Glioblastoma	3482 (80.6)
	Other	71(1.6)

### Regional Trends in Race Reporting

**Figure 3.**
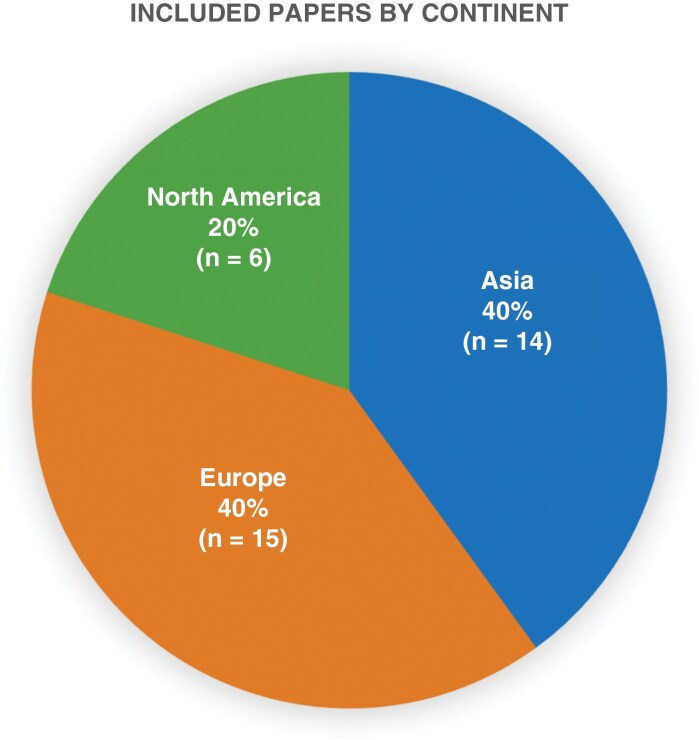
Pie chart demonstrating papers reviewed by continent (*n* = 35 studies).

**Figure 4.**
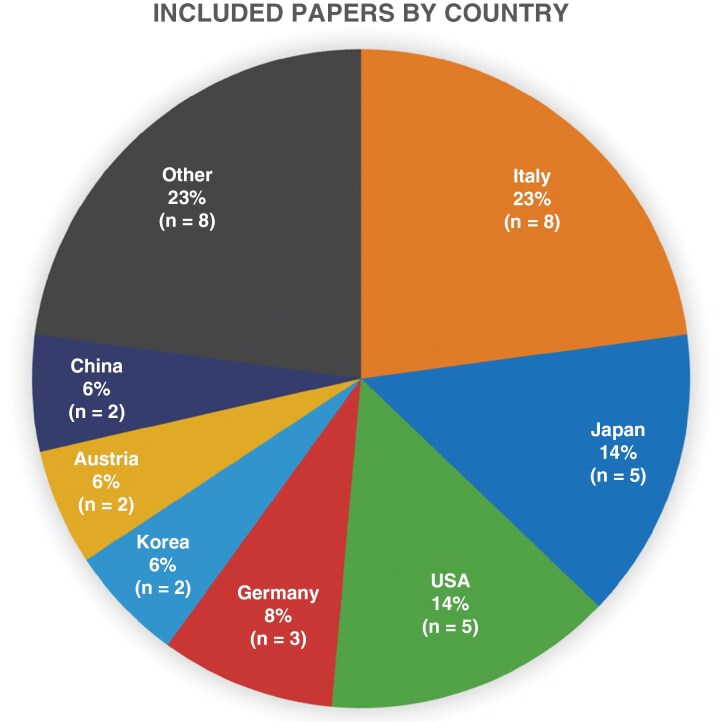
Pie chart demonstrating papers reviewed by country (*n* = 35 studies).

Of the 35 studies that met our inclusion criteria, including reporting de novo genome-scale/omic data generated from patient-derived samples, all studies were led by authors in one of 3 continents: Europe (15 studies, 43.0%), Asia (14 studies, 40.0%), and North America (6 studies, 17.1%), with no eligible studies from other continents including Africa and Australia (Figure 3). The countries most represented were the United States with 5 studies, Japan with 5 studies, and Italy with 8 studies (Figure 4). Of all 35 studies analyzed, only 2 studies reported on race (5.7% of studies) and only 1 reported on ethnicity (2.9% of studies). Both studies reporting on race were based in the United States while the study reporting on ethnicity was written by authors in Mexico. Therefore, reporting of race in glioma studies with de novo genome-scale/omic data was found to be higher in North American studies (2 out of 5 studies, 40.0%), compared to studies from Asia (0 out of 14 studies), and Europe (0 out of 15 studies). None of the papers made it possible to stratify individual patient or sample level data by race or ethnic group.

### Race and Ethnicity Reporting

**Figure 5.**
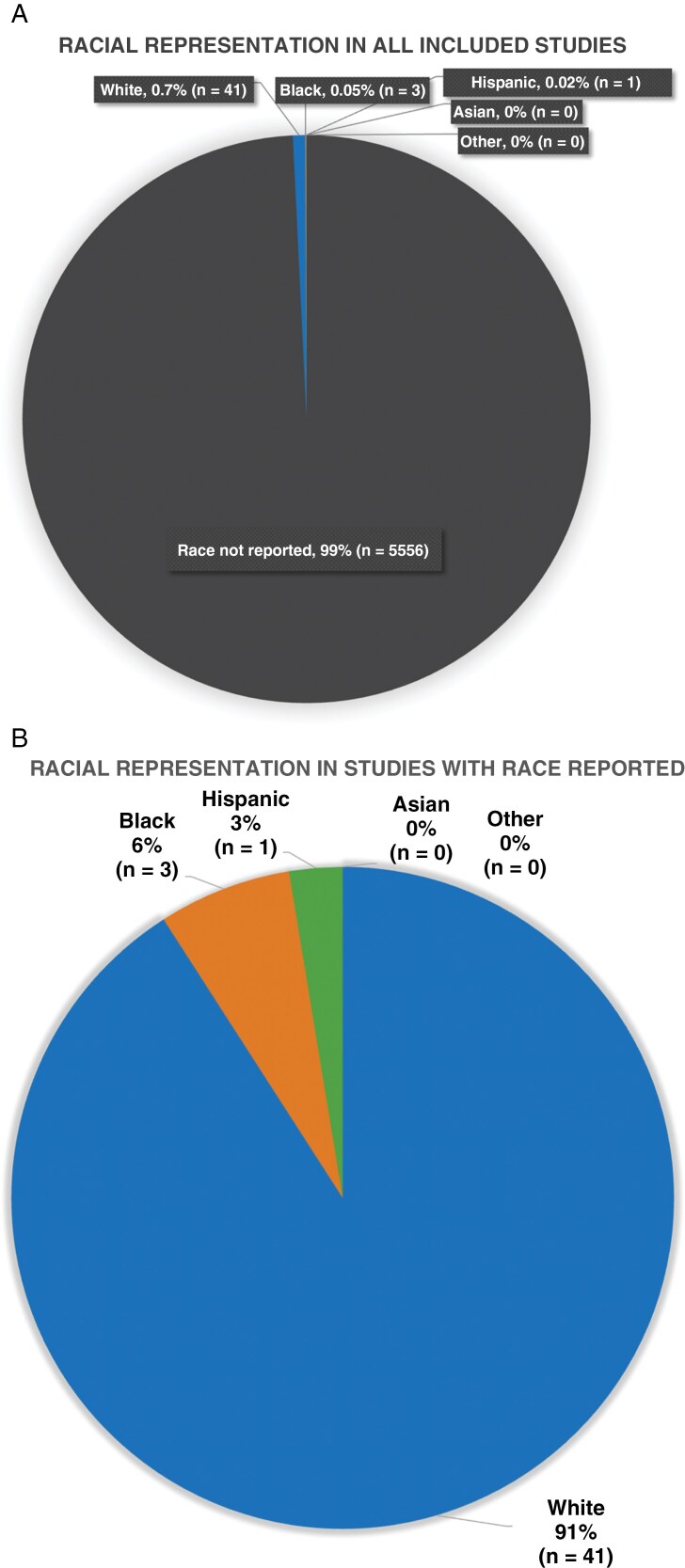
(A) Pie chart demonstrating the reporting of racial and ethnic makeup of participants included in analyzed studies (*n* = 5601 participants). (B) Pie chart demonstrating racial makeup of participants whose race was reported in analyzed papers (*n* = 45 participants).

The 35 papers analyzed had a total of 5601 patients. Race was described for 45 of those patients. Of those, 91.1% of patients with race reported were White, 6.7% were Black, and 2.2% were Hispanic. There were no Asian or people of “other race” identified in analyzed studies (Figure 5A and B).

## Discussion

Gliomas are tumors that originate from the glial cells of the CNS and represent over 80% of malignant brain tumors.^60,61^ They are graded from low grade (WHO grades 1 and 2) to high-grade (WHO grades 3 and 4). Glioblastoma (WHO grade 4) is the most common and aggressive form of primary brain tumor in adults and is typically known for its rapid progression and poor prognosis. Although gliomas are heavily studied in both preclinical and clinical research, with the expanding generation of omic datasets poised to continue supporting advances in biological understanding and guide the development of new treatment approaches, little data exists regarding racial and ethnic representation in the generation of such datasets. We, therefore, conducted a systematic review to provide a contemporary snapshot of current practices in the reporting of racial and ethnic information in glioma studies generating de novo genome-scale molecular data, given the importance of these rich molecular datasets within ongoing efforts to develop more effective treatments.

Our findings highlight an important and concerning trend in the lack of racial and ethnic representation in genomic studies of glioma. Only 2 studies (5.7%) analyzed included racial data, and just one study (2.8%) reported on ethnicity. This lack of routinely reporting racial and ethnicity data in studies generating “omic” data makes it impossible to accurately quantify how underrepresented certain groups might be and has important implications for attempts to generalize knowledge from these studies around the understanding and treatment of gliomas to all populations. This is particularly pertinent given recent confirmation that the distribution of brain tumor types, based on DNA methylation profiling, differs significantly across racial groups.^62^ We find that, in the small number of studies where race was reported, most of the participants were White (91.1%), with only 6.7% being Black and 2.2% Hispanic. No participants were reported as Asian or from other racial groups. Issues around a lack of race reporting were even more pronounced in studies conducted outside North America, with no studies from Asia or Europe reporting racial data. Additionally, there were no studies eligible for inclusion from other continents including Australia and Africa, highlighting the potential for ongoing underrepresentation of patients from these continents within the global repertoire of glioma omic datasets.

Gliomas, like many other cancers, exhibit genetic variations that can influence disease progression and response to treatment. By failing to include diverse populations in genomic studies, the neuro-oncology research community risks missing crucial genetic or other molecular markers that could lead to more effective, personalized treatments for glioma patients from a diverse range of backgrounds.^63,64^The overwhelming predominance of White patients in the studies we have examined may limit the potential of important study findings to be generalizable to other racial groups. Importantly, although we only examined studies which generated new omic data, excluding reanalysis of previously published omic data, the generation of new omic datasets and further studies reanalyzing these datasets is becoming increasingly prevalent within the neuro-oncology literature. Therefore, the impact of extremely limited race reporting and racial diversity in these contemporary studies is likely to directly influence future studies published for many years to come. Ultimately, this can result in treatments, treatment protocols and guidelines that are developed without adequate representation, or consideration of non-White populations and might be less effective in these contexts. Failure to represent these groups remains a current, collective and institutional challenge within the neuro-oncology research community, and could potentially exacerbate existing disparities in healthcare. Efforts must be made to include more a diverse range of participants in research to better reflect wider populations, and to fully understand and address the complex factors that influence health outcomes across different racial and ethnic groups. Certain genetic mutations such as those in *IDH1/2* genes are known to influence diagnosis, prognosis, and treatment response in glioma patients. If these mutations are more or less prevalent across different racial or ethnic groups, it is essential to identify and understand these differences, and understand if the biological impact of specific molecular changes varies between racial groups, to optimize treatment and guide prognosis for all.^65^

The current trend of underrepresentation means that potentially significant genetic insights are being overlooked, which could hinder the development of targeted therapies that are effective for all patient populations. This skewed representation in omic studies might also bias which therapeutic approaches are investigated within the context of clinical trials and lead to treatments that are developed based on data from predominantly White populations, which may not be as effective in patients from other racial backgrounds.^66,67^This can perpetuate existing health disparities and limit the efficacy of new therapies in diverse patient groups. To address these issues, it is crucial to implement measures that promote the inclusion of diverse populations in genomic research.^66^ Funding agencies and research institutions should prioritize diversity in study designs and participant recruitment as they have started to with regard to gender. Additionally, researchers should be encouraged to report racial and ethnic data comprehensively to ensure that the findings are applicable to a broader patient population. To improve this, we propose that minimum patient demographic reporting requirements incorporating race or ethnicity data (in addition to age, sex, and geographic information) should be incorporated into commonly used reporting checklists including those associated with CONSORT (Consolidated Standards of Reporting Trials) and STROBE (Strengthening the Reporting of Observational Studies in Epidemiology) guidelines for clinical studies and ARRIVE (Animal Research: Reporting of In Vivo Experiments) guidelines for preclinical studies, where the use of patient-derived samples are used (eg, in xenograft models).^68^ The limited reporting of race and ethnicity data, particularly in studies utilizing previously collected (including biobanked) tissue, may reflect a lack of routine recording or incomplete recording of this information in the clinical setting. We would therefore recommend joint discussion between key neuro-oncology societies, including SNO (Society for Neuro-Oncology), EANO (European Association of Neuro-Oncology), ASNO (Asian Society for Neuro-Oncology), BNOS (British Neuro-Oncology Society), SNO-SSA (Society for Neuro-Oncology Sub-Saharan Africa), and SNOLA (Society for Neuro-Oncology Latin America) to harmonize the brief, core data, and options that should be used to describe race and ethnicity within neuro-oncology studies, potentially using a World Federation of Neuro-Oncology Societies (WFNOS) meeting as a forum for these activities. This would help establish a global consensus on how race and ethnicity data is collected and integrated into both the recording of clinical information in routine practice and research to enable consistent comparisons between studies internationally. This well-respected forum would be ideally positioned to influence data recording, reporting, and initiatives to enhance inclusivity through member organizations, and apply pressure to organizations and initiatives such as the EQUATOR Network to promote the inclusion of standardized race and ethnicity reporting within study reporting checklists. We would also advocate for organizations funding research to mandate the inclusion of race and ethnicity data recording and reporting in studies which include patients as participants and/or use patient-derived materials (including tissue, animal xenografts, and cell lines). Additionally, within many existing, well-utilized omic datasets for gliomas where the reporting of race and ethnicity data is limited but obtainable, we would advocate research funders providing modest additional financial support to enable researchers to retrospectively increase the reporting of race and ethnicity information within existing datasets (through searching clinical records and/or contacting patients and families in a sensitive way, where feasible) to maximize the broader relevance and value of these resources.

The landscape of glioma research is undergoing a paradigm shift, with advancements in genomic sequencing offering unparalleled insights into tumor biology and potential therapeutic targets. However, an important piece of this puzzle remains largely absent: comprehensive data on patient race and ethnicity. This omission hinders our ability to deliver truly equitable and personalized medicine. Through this study, we have identified gaps in current research with the lack of diversity in patient representation in glioma studies generating omic datasets. This suggests that recently highlighted issues with racial representation in clinical trials^69^ also extend to preclinical studies. With this information, it is imperative to find new ways to increase the representation of patient populations in future preclinical studies and clinical trials to adequately understand how we can treat gliomas more effectively across all patient populations. An important factor to improve the lack of diversity would be aiming efforts at removing enrollment barriers for patients. Underrepresented patients tend to have higher enrollment barriers when compared to their represented counterparts. These barriers can include issues around transportation, information and awareness, compensation, communication, and loss to follow-up visits. Addressing each of these barriers can be crucial to enrolling more underrepresented patients in clinical trials.^70^ The U.S. Food and Drug Administration currently provides guidance for clinical investigators called *Payment and Reimbursement to Research Subjects,* a protocol that provides guidance for clinical trials on reimbursement for patients. Since financial burden (including due to missed work and fees associated with transportation) is often a reason patients are not able to participate in research, adherence to these guidelines are imperative and helpful in reducing financial barriers associated with enrolling in clinical studies. However, given the more preclinical nature of many studies using patient-derived samples to generate genome-scale datasets, our studies highlight an important need for these same principles of reimbursement and barrier reduction to be applied in contexts where patients may have the opportunity to donate tumor tissue for research use outside of a clinical trial. Furthermore, improving both physicians’ and patients’ awareness of research participation opportunities is important.

Interventions aimed at keeping physicians informed of opportunities for patient participation in research may be helpful. In parallel, efforts by researchers to collaborate with a diverse range of community groups and target clinics where patients already have trusted relationships would help enhance representation in future studies generating omic data for glioma patients. Creating collaborations with community groups or community health centers can lead to finding the best ways to reach and educate certain populations about ongoing clinical trials.^71^ Other recommendations include improving quality communication across the patient-physician relationship. Quality communication would be aimed at helping patients understand tissue use with preclinical studies and clinical trials, and the importance and positive impact of their contribution. Language, knowledge, and comfortability can be barriers to optimal communication during patient visits, therefore initiatives to reduce these communication barriers between physicians or researchers and patients are vital to support broader representation in research participation. For example, efforts to apply translator technology where a language barrier exists and using the teach-back method could be helpful if a knowledge or understanding barrier is suspected when discussing study participation with patients. In a linguistic analysis study that examined oncologist communication^72^ they found that interactions with African American patients were shorter when compared with White patients. They also found that the concept of clinical trials was discussed less frequently, and for a shorter duration when discussed, during these visits.

Although the rationale for these differences is likely to be complex and multifactorial, it is possible that interventions to tackle the potential unconscious bias of physicians and researchers involved in patient recruitment would improve representation in glioma research, including studies generating genome-scale datasets. Furthermore, the implementation of communication aids or “primers” incorporating rich-media such as a QR-code activated video animation might support patients from a range of backgrounds in making well-informed decisions around research participation.^73^ Lastly, initiatives can be aimed at creating pipeline programs to academic centers for underrepresented populations. Many underrepresented populations are more likely to present to under-resourced hospitals or less academic centers that may not have as many clinical studies or the infrastructure to support large research programs, including studies applying relatively costly omic sequencing technologies.^74^ Creating pipeline programs that direct patients toward academic centers could improve the overall inclusion and retention rates of underrepresented patient populations in future studies generating genome-scale molecular characterization of patient-derived glioma samples.

## Study Limitations

A limitation of our study is that it aimed to capture only a snapshot of literature between January and November of 2023. This was done to investigate the current state of racial and ethnic representation in sequencing of glial tumors in studies using the most recent WHO 2021 classification of CNS tumors. This approach means we were unable to look at trends in race and ethnicity reporting over time.

Another limitation is that only articles in English were included in this study, hence the literature in other languages will not be addressed in this review. In addition, our search strategy (*above*) might have incompletely captured omic studies focused exclusively on low grade gliomas.

Furthermore, whilst we carefully examined all included papers and their supplementary materials for race and ethnicity data reporting, we did not directly contact the authors of papers to establish whether they had unpublished race or ethnicity data available to share for their published cohorts. Nevertheless, we emphasize the need for race and ethnicity data associated with omic datasets to be publicly available, and easily accessible to maximize the impact of this important information, including through future secondary analyses and meta-analyses.

## Conclusion

This systematic review highlights extremely limited reporting and significant disparities in racial and ethnic representation within glioma genetic sequencing studies. These gaps underscore an urgent need for enhanced reporting and diversity to ensure equitable advancements in precision medicine. We strongly recommend that all future studies consistently report the race and ethnicity of patients providing samples, and that minimum demographic requirements including race and ethnicity data are added to commonly used reporting checklists. Addressing these critical issues will be pivotal in advancing healthcare equity in glioma research and ensuring that genomic discoveries benefit all populations.

## Supplementary material

Supplementary material is available online at *Neuro-Oncology Practice* (https://academic.oup.com/nop/).

npaf016_suppl_Supplementary_Material

## Data Availability

Study raw data will be made available online at *Neuro-Oncology** Practice*.
