# Supplementary material for: Race and “omic” data in glioma: A systematic review of contemporary research to explore the digital divide
Source: Neurooncol Pract. 2025 Jan 31;12(4):585–99. doi: 10.1093/nop/npaf016 (PMC12349769; doi:10.1093/nop/npaf016)
Supplement: npaf016_suppl_Supplementary_Material [file npaf016_suppl_supplementary_material.docx]

**Supplementary Table**

**Supplementary Table:** Search strategy

| **Database** | **Search Strategy** | **How many papers** |
| --- | --- | --- |
| Embase | (‘glioblastoma*’ OR ‘GBM’  OR ((‘high-grade’ OR ‘high grade’ OR ‘grade III’ OR ‘grade 3’ OR ‘grade IV’ OR ‘grade 4’) AND (‘glioma*’ OR ‘astrocytoma*’ OR ‘oligodendroglioma*’ OR ‘oligoastrocytoma*’)) OR ‘anaplastic astrocytoma*’ OR ‘anaplastic oligodendroglioma*’ OR ‘anaplastic oligoastrocytoma*’):ti,ab,kw  AND  (‘sequence analy*’ OR ‘deoxyribonucleic acid sequenc*’ OR ‘DNA sequenc*’ OR ‘protein sequenc*’ OR ‘exome sequenc*’ OR ‘genome sequenc*’ OR ‘genomic sequenc*’ OR ‘next generation sequenc*’ OR ‘next-generation sequenc*’ OR ‘mutational analy*’ OR ‘mutation analy*’ OR ‘multilocus sequence typ*’ OR ‘deep sequenc*’ OR ‘high throughput nucleotide sequenc*’ OR ‘high-throughput nucleotide sequenc*’ OR ‘chromatin immunoprecipitation sequenc*’ ‘Assay for Transposase-Accessible Chromatin’ OR ‘ribosome profil*’ OR ‘ribonucleic acid-sequenc*’ OR ‘ribonucleic acid sequenc*’ OR ‘RNA-seq*’ OR ‘RNA sequenc*’ OR ‘molecular sequence annotat*’ OR ‘nanopore sequenc*’ OR ‘oligonucleotide sequenc*’ OR ‘oligonucleotide array sequenc*’ OR ‘DNA barcod*’ OR ‘DNA contaminat*’ OR ‘deoxyribonucleic acid contamination*’ OR ‘peptide map*’ OR ‘massively parallel sequenc*’ OR ‘massively-parallel sequenc*’ OR ‘pyrosequenc*’ OR ‘single cell sequenc*’ OR ‘transcriptome sequenc*’ OR ‘methylome sequenc*’  ):ti,ab,kw | 400 |
| Scopus | (“glioblastoma*” OR “GBM”  OR ((“high-grade” OR “high grade” OR “grade III” OR “grade 3” OR “grade IV” OR “grade 4”) AND ( “glioma*” OR “astrocytoma*” OR “oligodendroglioma*” OR “oligoastrocytoma*”)) OR “anaplastic astrocytoma*” OR “anaplastic oligodendroglioma*” OR “anaplastic oligoastrocytoma*”)  AND  (“sequence analy*” OR “deoxyribonucleic acid sequenc*” OR “DNA sequenc*” OR “protein sequenc*” OR “exome sequenc*” OR “genome sequenc*” OR “genomic sequenc*” OR “next generation sequenc*” OR “next-generation sequenc*” OR “mutational analy*” OR “mutation analy*” OR “multilocus sequence typ*” OR “deep sequenc*” OR “high throughput nucleotide sequenc*” OR “high-throughput nucleotide sequenc*” OR “chromatin immunoprecipitation sequenc*” “Assay for Transposase-Accessible Chromatin” OR “ribosome profil*” OR “ribonucleic acid-sequenc*” OR “ribonucleic acid sequenc*” OR “RNA-seq*” OR “RNA sequenc*” OR “molecular sequence annotat*” OR “nanopore sequenc*” OR “oligonucleotide sequenc*” OR “oligonucleotide array sequenc*” OR “DNA barcod*” OR “DNA contaminat*” OR “deoxyribonucleic acid contamination*” OR “peptide map*” OR “massively parallel sequenc*” OR “massively-parallel sequenc*” OR “pyrosequenc*” OR “single cell sequenc*” OR “transcriptome sequenc*” OR “methylome sequenc*”) | 84 |
| Web of Science | TS=  (“glioblastoma*” OR “GBM” OR ((“high-grade” OR “high grade” OR “grade III” OR “grade 3” OR “grade IV” OR “grade 4”) AND (“glioma*” OR “astrocytoma*” OR “oligodendroglioma*” OR “oligoastrocytoma*”)) OR “anaplastic astrocytoma*” OR “anaplastic oligodendroglioma*” OR “anaplastic oligoastrocytoma*”)  AND  (“sequence analy*” OR “deoxyribonucleic acid sequenc*” OR “DNA sequenc*” OR “protein sequenc*” OR “exome sequenc*” OR “genome sequenc*” OR “genomic sequenc*” OR “next generation sequenc*” OR “next-generation sequenc*” OR “mutational analy*” OR “mutation analy*” OR “multilocus sequence typ*” OR “deep sequenc*” OR “high throughput nucleotide sequenc*” OR “high-throughput nucleotide sequenc*” OR “chromatin immunoprecipitation sequenc*” “Assay for Transposase-Accessible Chromatin” OR “ribosome profil*” OR “ribonucleic acid-sequenc*” OR “ribonucleic acid sequenc*” OR “RNA-seq*” OR “RNA sequenc*” OR “molecular sequence annotat*” OR “nanopore sequenc*” OR “oligonucleotide sequenc*” OR “oligonucleotide array sequenc*” OR “DNA barcod*” OR “DNA contaminat*” OR “deoxyribonucleic acid contamination*” OR “peptide map*” OR “massively parallel sequenc*” OR “massively-parallel sequenc*” OR “pyrosequenc*” OR “single cell sequenc*” OR “transcriptome sequenc*” OR “methylome sequenc*”) | 330 |
| PubMed | ("glioblastoma"[MeSH] OR "glioblastoma*"[tiab] OR "GBM"[tiab])  OR (("high-grade"[tiab] OR "high grade"[tiab] OR "grade III"[tiab] OR "grade 3"[tiab] OR "grade IV"[tiab] OR "grade 4"[tiab] OR "glioma"[MeSH]) AND ("astrocytoma"[MeSH] OR "oligodendroglioma"[MeSH] OR "glioma*"[tiab] OR "astrocytoma*"[tiab] OR "oligodendroglioma*"[tiab] OR "oligoastrocytoma*"[tiab] OR "anaplastic astrocytoma*"[tiab] OR "anaplastic oligodendroglioma*"[tiab] OR "anaplastic oligoastrocytoma*"[tiab]))  AND ("sequence analysis"[MeSH] OR "exome sequencing"[MeSH] OR "whole genome sequenc*"[MeSH] OR "high-throughput nucleotide sequencing"[MeSH] OR "DNA mutational analysis"[MeSH] OR "DNA Barcoding, Taxonomic"[MeSH] OR "Multilocus Sequence Typing"[MeSH] OR "sequence analy*"[tiab] OR "deoxyribonucleic acid sequenc*"[tiab] OR "DNA sequenc*"[tiab] OR "protein sequenc*"[tiab] OR "exome sequenc*"[tiab] OR "genome sequenc*"[tiab] OR "genomic sequenc*"[tiab] OR "next generation sequenc*"[tiab] OR "next-generation sequenc*"[tiab] OR "mutational analy*"[tiab] OR "mutation analy*"[tiab] OR "multilocus sequence typ*"[tiab] OR "deep sequenc*"[tiab] OR "high throughput nucleotide sequenc*"[tiab] OR "high-throughput nucleotide sequenc*"[tiab] OR "chromatin immunoprecipitation sequenc*"[tiab] OR "Assay for Transposase-Accessible Chromatin"[tiab] OR "ribosome profil*"[tiab] OR "ribonucleic acid-sequenc*"[tiab] OR "ribonucleic acid sequenc*"[tiab] OR "RNA-seq*"[tiab] OR "RNA sequenc*"[tiab] OR "molecular sequence annotat*"[tiab] OR "nanopore sequenc*"[tiab] OR "oligonucleotide sequenc*" OR "oligonucleotide array sequenc*"[tiab] OR "DNA barcod*"[tiab] OR "DNA contaminat*"[tiab] OR "deoxyribonucleic acid contamination*"[tiab] OR "peptide map*"[tiab] OR "massively parallel sequenc*"[tiab] OR "massively-parallel sequenc*"[tiab] OR "pyrosequenc*"[tiab] OR "single cell sequenc*"[tiab] OR "transcriptome sequenc*"[tiab] OR "methylome sequenc*"[tiab]) | 445 |
